# Multiscale analysis of heart rate variability in non-stationary environments

**DOI:** 10.3389/fphys.2013.00119

**Published:** 2013-05-30

**Authors:** Jianbo Gao, Brian M. Gurbaxani, Jing Hu, Keri J. Heilman, Vincent A. Emanuele II, Greg F. Lewis, Maria Davila, Elizabeth R. Unger, Jin-Mann S. Lin

**Affiliations:** ^1^PMB Intelligence LLCWest Lafayette, IN, USA; ^2^Mechanical and Materials Engineering, Wright State UniversityDayton, OH, USA; ^3^Chronic Viral Diseases Branch, Division of High Consequence Pathogens and Pathology, Centers for Disease Control and PreventionAtlanta, GA, USA; ^4^College of Medicine, Brain-Body Center, University of IllinoisChicago, IL, USA; ^5^Research Triangle InstituteRaleigh, NC, USA

**Keywords:** heart rate variability, fractal, adaptive fractal analysis, chaos, scale-dependent Lyapunov exponent, Trier Social Stress Test, chronic fatigue syndrome

## Abstract

Heart rate variability (HRV) is highly non-stationary, even if no perturbing influences can be identified during the recording of the data. The non-stationarity becomes more profound when HRV data are measured in intrinsically non-stationary environments, such as social stress. In general, HRV data measured in such situations are more difficult to analyze than those measured in constant environments. In this paper, we analyze HRV data measured during a social stress test using two multiscale approaches, the adaptive fractal analysis (AFA) and scale-dependent Lyapunov exponent (SDLE), for the purpose of uncovering differences in HRV between chronic fatigue syndrome (CFS) patients and their matched-controls. CFS is a debilitating, heterogeneous illness with no known biomarker. HRV has shown some promise recently as a non-invasive measure of subtle physiological disturbances and trauma that are otherwise difficult to assess. If the HRV in persons with CFS are significantly different from their healthy controls, then certain cardiac irregularities may constitute good candidate biomarkers for CFS. Our multiscale analyses show that there are notable differences in HRV between CFS and their matched controls before a social stress test, but these differences seem to diminish during the test. These analyses illustrate that the two employed multiscale approaches could be useful for the analysis of HRV measured in various environments, both stationary and non-stationary.

## 1. Introduction

Modern interest in heart rate variability (HRV) began when it was observed that it is more than an important and easily accessible indicator of cardiovascular function, but an important measure of autonomic nervous system (ANS) function and health in general. A salient feature is its spontaneous fluctuation, even if the environmental parameters are maintained constant and no perturbing influences can be identified. Obviously, variations in HRV will be more complicated if the data are measured in intrinsically non-stationary environments. Therefore new methods for better characterizing HRV measured in those situations are desirable.

Since the first observations that HRV can be a sensitive indicator of declining health that precedes changes in heart rate itself or other physiological measures of distress (Hon and Lee, 1963; Kleiger et al., 1987; King et al., 2009), a number of methods have been proposed to analyze HRV data. The most widely used methods assume a stationary process underlying the statistics, calculated from time and frequency domain analyses [see Malik (1996) and references therein], as well as those derived from chaos theory and random fractal theory (Kobayashi and Musha, 1982; Goldberger and West, 1987; Babyloyantz and Destexhe, 1988; Kaplan and Goldberger, 1991; Pincus and Viscarello, 1992; Bigger et al., 1996; Ho et al., 1997). In this paper, we illustrate the general use of two multiscale approaches that do not assume a stationary process, the *adaptive fractal analysis* (AFA) (Gao et al., 2011b; Kuznetsov et al., 2012; Riley et al., 2012), and the *scale-dependent Lyapunov exponent* (SDLE) (Gao et al., 2006a, 2007, 2012d), for the analysis of HRV in a study of chronic fatigue syndrome (CFS) patients with matched healthy controls.

Multiscale analysis of heart rate was first considered over a decade ago by groups exploring non-linear dynamics in physiology, for example (Costa et al., 2002, 2005). These studies, while quite interesting in their methods, explored datasets that were not subtle in their impact on cardiovascular physiology. Such sophisticated techniques were not needed to distinguish healthy heart rates from those in the diseases being considered (congestive heart failure and atrial fibrillation). The same dataset has been explored further by many others using different multiscale analysis techniques, for example (Hu et al., 2009a, 2010). These analyses have added to a growing interest in multiscale analysis of physiology in general (West, 2010), but studies using multiscale techniques to analyze more challenging datasets where the cardiac disturbances are more subtle (such as those found in CFS) are very rare. The techniques demonstrated here show some promise when the cardiac disturbances are subtle and the effect sizes are small.

CFS is a heterogeneous illness, with subsets of patients potentially having autonomic nervous system (ANS), immune system, and endocrine system involvement (Newton et al., 2011; Rahman et al., 2011). While no known biomarker for CFS has been identified to date, recent small-sample studies have suggested certain cardiac irregularities, such as low nocturnal HRV (Boneva et al., 2007; Burton et al., 2009; Rahman et al., 2011), small left ventricle and orthostatic intolerance (Miwa and Fujita, 2011), short QT interval (Naschitz et al., 2006), and low blood volume and diminished cardiac function (Hurwitz et al., 2009; Hollingsworth et al., 2011), are associated with CFS. It is not known if the cardiac irregularities are themselves a cause of the symptoms of CFS or if they are symptoms of the autonomic dysfunction and other systemic breakdowns which are more directly causative. Whatever the case, analysis of ECG data appears to show promise and, given the data accessibility and feasibility of collecting it, it behooves us to look beyond resting data to data collected in more stressful environments, and to analyze the data with methods that do not assume stationarity.

We shall employ two relatively new and fundamentally different multiscale approaches to characterize HRV of control and CFS patients. While we hope to shed some new light on the pathology of CFS through analysis of HRV, for the interest of this special issue, we will make our best efforts to illustrate how these methods are used to analyze our data, so that interested readers may readily adapt the methods to analyze their HRV data measured in other situations, both stationary and non-stationary.

## 2. Methods

### 2.1. Data

The HRV data analyzed here were derived from subjects who participated on the third day of the 3-day clinical study taking place at Emory University's Atlanta Clinical and Translational Science Institute (ACTSI, formerly known as General Clinical Research Center or GCRC). Subjects who participated in the ACTSI study were identified from the baseline (2004–2005) (Reeves et al., 2005) and follow-up waves of the Georgia longitudinal study on CFS and Chronic Unwellness. This study adhered to human experimentation guidelines of the U.S. Department of Health and Human Services, was approved by the Centers for Disease Control and Prevention (CDC) Human Subjects Review Board, and all participants gave written informed consent. The ACTSI study was a 1:2 case-control study design with matching on age within 5 years, sex, race/ethnicity, and BMI (≥30 kg/m^2^, obese or not). The case-control status was determined by the classifications from the two waves (baseline and follow-up) of the Georgia longitudinal study. Subjects stayed overnight at the Emory clinic during their participation in the 3-day ACTSI study. The study consisted of two components: the first 2 days involved brain imaging in conjunction with studies of cognition and the ANS, and the third day involved a stress-induced challenge V the Trier Social Stress Test (TSST) V to test the hypothalamic pituitary adrenal (HPA) axis, ANS, and immune system. Between March 2008 and July 2009, there were 36 CFS cases and 48 non-fatigued healthy controls completed the 3-day study. The current analysis will focus on 23 CFS and 41 controls with the HRV data collected during the Day-3 TSST study. Heart rate data were collected using Biopac feeds into Somnologica software at 200 Hz on a Dell laptop computer. A technician attached sensors to subjects and calibrated equipment prior to the TSST. Subjects wore the electrodes and the recording device from 1:30 pm to 4:00 pm on Day 3. The ECG data were then stored and transferred to CDC computers. R–R interval pre-processing from the raw ECG was done with a LabVIEW script developed at the Brain-Body Center at University of Illinois, Chicago. This processing interpolated the R peaks from the 200 Hz data to improve their temporal localization.

Event times were annotated at the clinic in the Somnologica software itself, or, occasionally, in an Excel spreadsheet when the ECG recorder and/or Somnologica software was malfunctioning. Twelve 5-min intervals were chosen to represent each subject during the roughly 3 h of the TSST. These intervals correspond to three events (e.g., blood draws) before the test, four key events during the test (the receptionist's speech, a blood draw, the subject's TSST speech, and the math test), and five events after the test (blood draws), and allow correlations with gene expression to be made. All IBI data (inter-beat-interval, also called R–R interval, time between R peaks in msec) were manually inspected and corrected for artifacts prior to analyses (CardioEdit; Brain-Body Center, University of Illinois at Chicago, 2007). Missed and/or incorrect R peak detections were manually corrected using the ECG waveform when available. Otherwise, artifacts were corrected using integer arithmetic (i.e., dividing intervals when detections were missed and adding intervals when spuriously invalid detections occurred). During this process, event times were checked for discrepancies between the Somnologica annotations and the master Excel spreadsheet recording for all event times, and between those recorded times and other data (e.g., physiological events like rapid rise in heart rate during the TSST speech). Not all subjects had interpretable, cleanable raw ECG data, or well marked event times for all 12 of the data intervals. Some subjects suffered from arrhythmias which made the R peak picking software unreliable, and their data could not be used in this study. Furthermore, we excluded six subjects who were not consistently classified as CFS at some point during the multi-year study. Thus, in this study we used a reduced subset of all the subjects who underwent the TSST. Losses to the subject pool skewed somewhat the sex, age, race, and BMI matching of the study, but not significantly as can be seen in Table 1 below (*p*-values for age and BMI computed with a 2-sided *t*-test; those for sex and race using a 2-tailed Fisher's exact test).

**Table 1:** **Deomgraphics of subjects used in this study**.

	**CFS (23)**	**Control (41)**	***p*-value**
Sex (% female)	91.3	75.6	0.18
Age (average)	47.6	46.3	0.57
Race (% white)	73.9	85.4	0.32
BMI (average)	28.3	26.8	0.21

**Figure 1:**
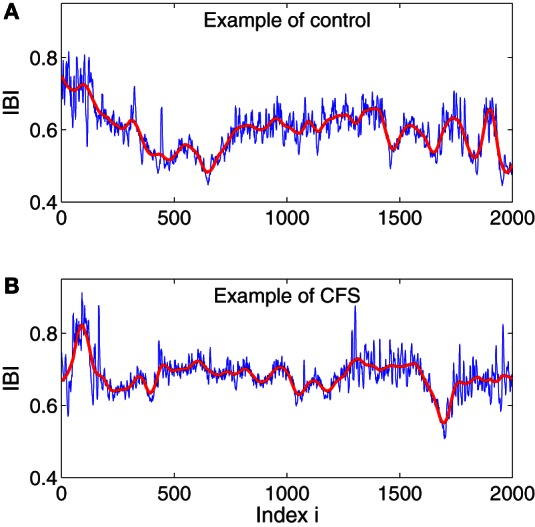
**Examples of IBI data for a control (A) and CFS (B) subject.** The red curves are the trend signals obtained by the adaptive filter, which will be explained below.

In this study, we will focus on the analysis of HRV data measured before and during the stress tests. To appreciate what our HRV data look like, we have shown in Figures 1A,B IBI data of a control and a CFS subject measured during the stress test. While the details of the two IBI data sets are different, we do observe some statistical similarity between them.

### 2.2. Adaptive fractal analysis (AFA)

In this paper we will characterize the fractal nature of the IBI time series with the Hurst parameter, *H*. Although many excellent methods for estimating *H* exist, including the famous detrended fluctuation analysis (Peng et al., 1994), care should be exercised by their interpretation, particularly if one is faced with relatively short time series that contain trends, non-stationarity, or signs of rhythmic activity (Hu et al., 2009b). One of the better approaches to tackle these difficulties is the AFA (Gao et al., 2011b, 2012a; Kuznetsov et al., 2012; Riley et al., 2012). In the following, we will attempt to explain the method clearly as we apply it to HRV data.

AFA is based on non-linear adaptive multiscale decomposition (Gao et al., 2010; Tung et al., 2011), which starts by partitioning a time series into segments of length *w* = 2*n* + 1, where neighboring segments overlap by *n* + 1 points, which ensures symmetry. Each segment is then fitted with the best polynomial of order *M*. Note that *M* = 0 and 1 correspond to piece-wise constant and linear fitting, respectively. We denote the fitted polynomials for the *i*-th and (*i* + 1)-th segments by *y*^(*i*)^(*l*_1_) and *y*^(*i*+1)^(*l*_2_), respectively, where *l*_1_, *l*_2_ = 1, …, 2*n* + 1. We then define the fitting for the overlapped region as

(1) $$y^{\left(c\right)}(l)=w_{1}y^{\left(i\right)}(l+n)+w_{2}y^{\left(i+1\right)}(l),\;l=1,\;2,\;\ldots ,\;n+1,$$

where $w_{1}=(1-\frac{l-1}{n})$ and $w_{2}=\frac{l-1}{n}$ can be written as (1 − *d*_*j*_/*n*) for *j* = 1, 2, and where *d*_*j*_ denotes the distances between the point and the centers of *y*^(*i*)^ and *y*^(*i* + 1)^, respectively. This means that the weights decrease linearly with the distance between the point and the center of the segment. Such a weighting ensures symmetry and effectively eliminates any jumps or discontinuities around the boundaries of neighboring segments. In fact, the scheme ensures that the fitting is continuous everywhere, is smooth at the non-boundary points, and has the right- and left-derivatives at the boundary. Moreover, since it can deal with an arbitrary trend without *a priori* knowledge, it can remove non-stationarity, including baseline drifts and motion artifacts (Gao et al., 2011b), and the procedure may also be used as either high-pass or low-pass filter with superior noise-removal properties than linear filters, wavelet shrinkage, or chaos-based noise reduction schemes (Tung et al., 2011). In Figures 1A,B we have plotted two trend signals in red for the IBI data shown there, using a window size of *w* = 101 and a polynomial order of 2.

Based on the described adaptive decomposition, a fractal analysis can be conducted as follows. Denote IBI data as *u*(*i*), *i* = 1, …, *N*, and the global smooth trend such as shown as red curves in Figures 1A,B as *v*(*i*), *i* = 1, …, *N*. AFA essentially is a scaling law relating the variance of the residual time series *u*(*i*)−v(*i*)and the window size *w* (Gao et al., 2011b)

(2) $$F(w)={\left[\frac{1}{N}\sum_{i=1}^{N}{\left(u(i)-v(i)\right)}^{2}\right]}^{1/2}\sim w^{H}.$$

Note that in this formulation, IBI data are treated as random walk processes. This is consistent with the literature (Peng et al., 1993; Hu et al., 2010) and what is seen in Figures 1A,B. Also note that for truly fractal processes, the polynomial order does not matter. This is also largely true for IBI data. In this work, we always fix the polynomial order to be 1.

### 2.3. Scale-dependent lyapunov exponent (SDLE) analysis

SDLE is a multiscale complexity measure first introduced in 2006 (Gao et al., 2006a, 2007). It has been further developed theoretically (Gao et al., 2009, 2012b) and applied to characterize EEG (Gao et al., 2011a, 2012c), HRV (Hu et al., 2009a, 2010), financial time series (Gao et al., 2011c), and Earth's geodynamo (Ryan and Sarson, 2008). Recently, SDLE is compared with a number of entropy measures (Gao et al., 2012c). It is found that SDLE has superior scaling behaviors—it has well-defined scaling laws for all known major classes of time series, and embodies all the information approximate entropy and sample entropy may have. Since we have done an in depth tutorial of SDLE in Gao et al. (2012d), here we will only briefly describe SDLE is such a way that the material presented here is self-contained.

SDLE is a concept defined in a high-dimensional phase space using the time delay embedding technique (Packard et al., 1980; Takens, 1981; Sauer et al., 1991). This technique is perhaps the most significant contribution of chaos theory to practical data analysis, since non-trivial dynamical systems usually involve many state variables, and therefore have to be described by a high-dimensional state (or phase) space. Consider a scalar time series *x*[*n*] = *x*(1), *x*(2), …, *x*(*n*). The embedding technique consists of creating vectors of the form:

(3) $$\begin{array}{l} V_{i}=[x(i),\;x(i+L),\;\ldots ,\;x(i+(m-1)L)],\;i=1,\;\ldots ,\\ \;N_{p}=n-(m-1)L, \end{array}$$

where *n* is the total length of the time series, *N*_*p*_ is the total number of constructed vectors, *m* is the embedding dimension and *L* the delay time. The embedding parameters need to be chosen according to certain optimization criteria. For details, we refer to chapter 13 of our book Gao et al. (2007).

After a proper phase space is re-constructed, we consider an ensemble of trajectories. We denote the initial separation between two nearby trajectories by ε_0_, and their *average separation* at time *t* and *t* + Δ*t* by ε_*t*_ and ε_*t* + Δ*t*_, respectively. We can then examine the relation between ε_*t*_ and ε_*t* + Δ*t*_, where Δ*t* is small. When Δ*t* → 0, we have,

(4) $$\varepsilon_{t+\Delta t}=\varepsilon_{t}e^{\lambda (\varepsilon_{t})\Delta t},$$

where λ (ε_*t*_) is the SDLE given by

(5) $$\lambda (\varepsilon_{t})=\frac{\ln\varepsilon_{t+\Delta t}-\ln\varepsilon_{t}}{\Delta t}.$$

Equivalently, we can express this as,

(6) $$\frac{d\varepsilon_{t}}{dt}=\lambda (\varepsilon_{t})\varepsilon_{t}.$$

To compute SDLE, we check whether pairs of vectors (*V*_*i*_, *V*_*i*_) defined by Equation (3) satisfy the following *Inequality*,

(7) $$\varepsilon_{k}\leq \left\|V_{i}-V_{j}\right\|\leq \varepsilon_{k}+\Delta \varepsilon_{k},\;k=1,\;2,\;3,\;\ldots ,$$

where ε_*k*_ and Δε_*k*_ are arbitrarily chosen small distances, and

(8) $$||V_{i}-V_{j}||=\sqrt{\sum_{w=1}^{m}{\left(x_{i+(w-1)L}-x_{j+(w-1)L}\right)}^{2}}$$

Geometrically, *Inequality* (Equation 7) defines a high-dimensional shell (which reduces to a ball with radius Δε_*k*_ when ε_*k*_ = 0; in a 2-D plane, a ball is a circle described by (*x* − *a*)^2^ + (*y* − *b*)^2^ = *r*^2^, where (*a*, *b*) is the center of the circle, and *r* is the radius). We then monitor the evolution of all such vector pairs (*V*_*i*_, *V*_*j*_) within a shell and take the ensemble average over indices *i*, *j*. Since we are most interested in exponential or power-law functions, we assume that taking logarithm and averaging can be exchanged, then Equation (5) can be written as

(9) $$\lambda (\varepsilon_{t})=\;\frac{\langle \ln\|V_{i+t+\Delta t}-V_{j+t+\Delta t}\|-\ln\|V_{i+t}-V_{j+t}\|\rangle }{\Delta t}$$

where *t* and Δ*t* are integers in units of the sampling time, the angle brackets denote the average over indices *i*, *j* within a shell, and

(10) $$\varepsilon_{t}=\|V_{i+t}-V_{j+t}\|=\sqrt{\sum_{w=1}^{m}{\left(x_{i+(w-1)L+t}-x_{j+(w-1)L+t}\right)}^{2}}$$

To ease the following discussion, we now list two scaling laws for SDLE:

1. For *clean chaotic signal*, λ(ε) fluctuates slightly around a constant. As is expected, this constant is the very largest positive Lyapunov exponent, λ_1_,
  (11) $$\lambda (\varepsilon )=\lambda_{1}.$$
2. For noisy dynamics, on small scales,
  (12) λ(ε)~−γlnε,
  where γ is a coefficient controlling the speed of loss of information (i.e., defined as the measure of uncertainty involved in predicting the value of a random variable). This feature suggests that entropy generation is infinite when the scale ε approaches zero.

## 3. Results

### 3.1. AFA of HRV

Random fractal theory is perhaps the most famous model for HRV. A central theme of random fractal theory is the notion of long-range correlation characterized by the Hurst parameter *H* (Gao et al., 2006b, 2007): a time series is said to have anti-persistent, short-range or memoryless, or persistent long-range correlations if 0 < *H* < 1/2, *H* = 1/2, or 1/2 < *H* < 1, respectively. A classic result about HRV dynamics is that HRV data measured in quiet conditions possess anti-persistent correlations characterized by 0 < *H* < 1/2 (Peng et al., 1993).

**Figure 2:**
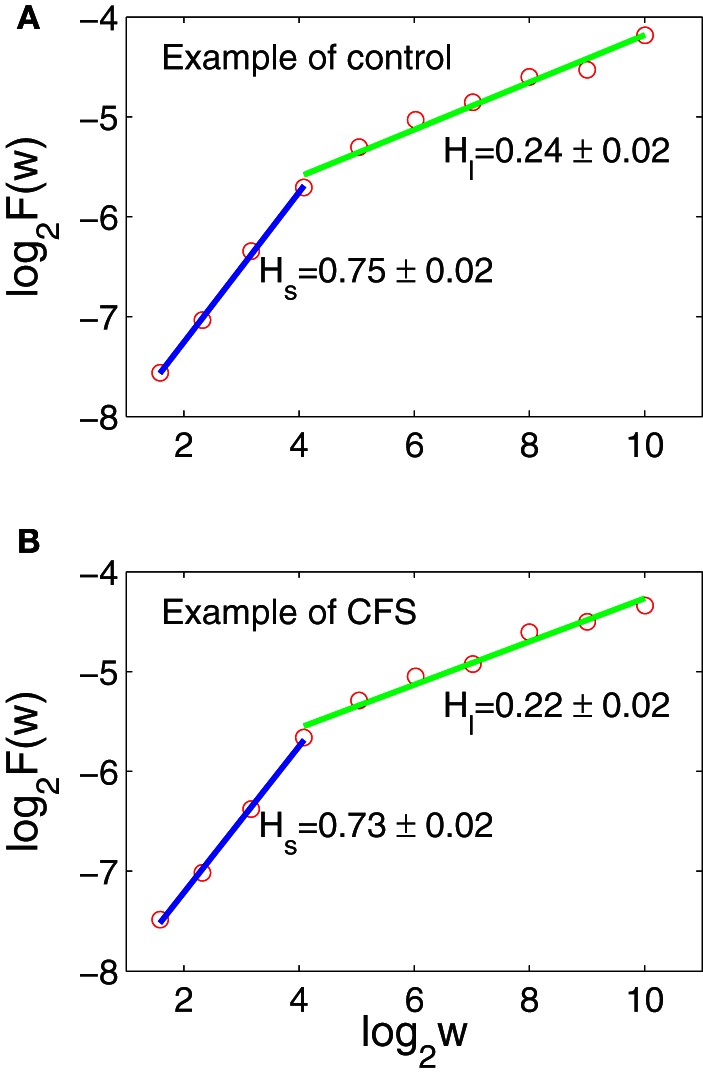
**AFA of the IBI data shown in Figures 1A,B.** The scaling break around *w* = 2^4^ is generic among all the subjects. **(A,B)** The red circles are for computed H, the blue lines are a linear fit for H_*s*_, and the green lines are linear fits for H_*l*_.

Figure 2 shows two AFA curves for the data shown in Figures 1A,B. We observe that the scaling breaks around *w* = 2^4^. It turns out this is a generic feature among all the subjects. Note that the Hurst parameter *H*_*s*_ for the short-time scale is larger than 1/2, different from most of the literature that *H* for HRV is usually smaller than 1/2. The difference could be because our IBI data were measured during the TSST, while most published results (e.g., Peng et al., 1993; Hu et al., 2010) used HRV data measured in resting state. Another explanation could be that on short time scales HRV is more persistent, as is suggested by Poincare plots of heart rate, which typically show a strong positive correlation (Otzenberger et al., 1998; Smith and Reynolds, 2006; Smith et al., 2007). None-the-less, we observe that *H*_*l*_ is smaller than 1/2, indicating that on long time scales, IBI data of control and CFS subjects still have anti-persistent correlations, similar to HRV data measured in resting states. Finally, we note that the two AFA curves are very similar. This is because the original IBI data are similar, as we pointed out earlier.

**Figure 3:**
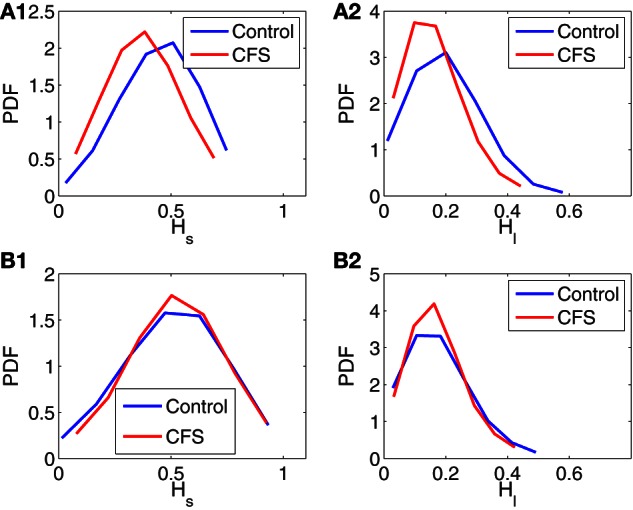
**Probability density functions (PDFs) for H_*s*_ and *H*_*l*_ of control and CFS subjects, where (A1, A2) are for data measured before the TSST, and (B1, B2) are for data measured during the stress tests**.

The two very desirable properties of AFA are (1) it can readily deal with arbitrarily complicated trends, and (2) it works well even when the data set is short (Gao et al., 2012a). The latter property enables us to apply AFA to the six 5-min IBI data measured before and during the TSST (3 before and 3 during the TSST). When AFA is extended to these data sets, according to the two conditions, before and during the TSST, we find that the knee point around *w* ≈ 2^4^ is still generically present. The second, long-time scale scaling regime, however, becomes shorter, since the IBI data are shorter. Nevertheless, *H*_*s*_ and *H*_*l*_ are still well-defined. The results are summarized in Figure 3, where (**A1,A2**) are for the data measured before the TSST, and (**B1,B2**) for the data measured during the stress tests. Simple statistical tests on the PDF's shown in Figure 3 are shown in Table 2. We observe that before the TSST, group differences exist for *H*_*s*_ and *H*_*l*_ between control and CFS subjects, although the area under the curve for the respective ROC plots indicates that each measure is only weakly discriminatory for individual subjects. However, neither *H*_*s*_ nor *H*_*l*_ appears to show any substantial differences between control and CFS during the stress tests (ROC curves were not computed during the TSST due to their low discriminatory power). These preliminary observations bear further investigation using studies with larger sample sizes.

**Table 2:** **Statistical tests on *H*_*s*_ and *H*_*l*_ before and during the TSST**.

	***H*_*s*_ pre**	***H*_*l*_ pre**	***H*_*s*_ TSST**	***H*_*l*_ TSST**
*p*-value	0.0001	0.0071	0.62	0.98
ROC AUC	0.65	0.64	−	−

### 3.2. SDLE of HRV

In this section, we will focus on whether SDLE shows promise identifying differences between HRV of healthy control and CFS subjects during the TSST. For the purpose of the SDLE analysis, we ensured that 20–30 min of continuous IBI data during the TSST was obtained rather than only 5 min intervals. Such long data are needed for SDLE analysis. A drawback is that due to the small sample size, estimating the PDFs for metrics derived from SDLE analysis is ill-posed—this is more doable with AFA, since AFA works with 5-min IBI data, and we have several different data intervals before and during the TSST. Therefore with SDLE, we will use scatter plots.

**Figure 4:**
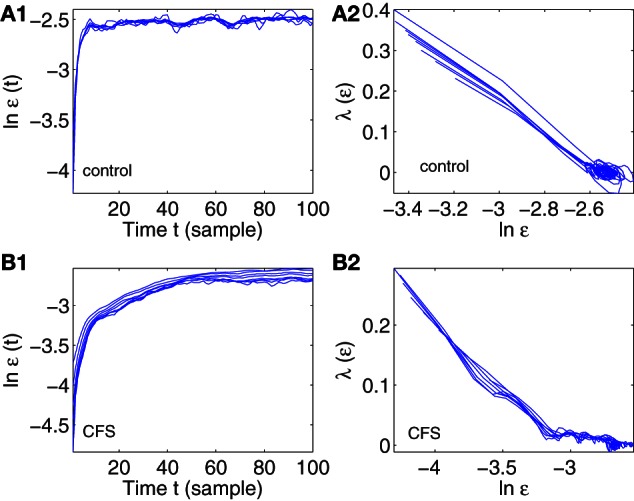
**Error growth ln ε(*t*) vs. *t* and SDLE λ(ε) vs. ln ε curves for a control (A1, A2) and CFS (B1, B2) subject**.

Figure 4 shows two examples of error growth curves, ln ε(*t*) vs. *t*, and their corresponding SDLE curves, for a control and a CFS subject. These examples were chosen to illustrate some of the differences between subjects with regards to SDLE, and do not illustrate general differences between CFS cases and healthy controls. We observe that the general dynamics on short time scales is noisy dynamics characterized by a rapid increase in ln ε(*t*), and a scaling of Equation 12. Such behavior holds for all the subjects.

**Figure 5:**
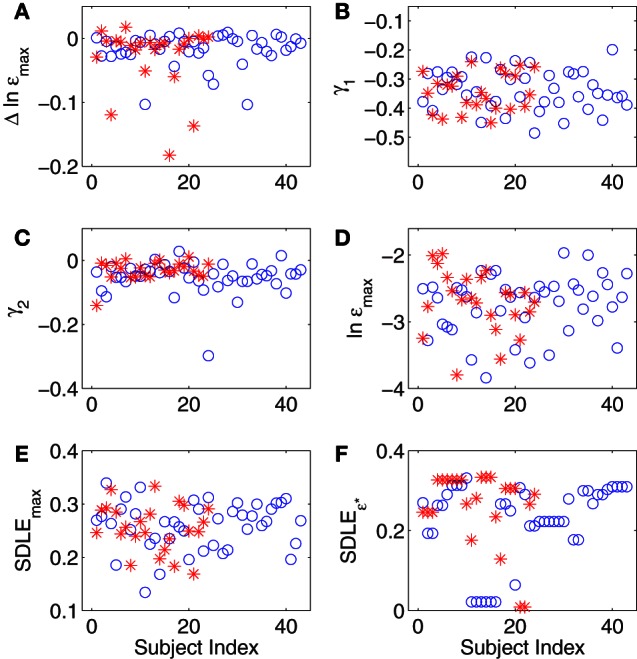
**(A–F)** Summary of SDLE analysis, where blue circles and red stars are for control and CFS subjects, respectively (see text for more details about each metric).

A number of metrics from the error growth and SDLE curves in Figure 4 can be derived and checked to determine if CFS and control groups can be separated in a statistically significant way. These metric are:

1. Δε_max_: It measures how far apart the error growth curves originating from different shells (or initial conditions) as shown in Figures 4A1,B1 are. It basically measured the degree of non-stationarity in the IBI data. The results are shown in Figure 5A.
2. The coefficient γ_1_ and γ_2_ of Equation (12), for small and large ε scales, respectively. Here, small ε generally refers to the range of ε where the SDLE curve is almost straight. They are ln ε ≤ −2.6 and ln ε ≤ −3.2 for Figures 4A2,B2, respectively. The large ε scales for those plots correspond to ln ε ≥ −2.6 and ln ε ≥ −3.2. The results are shown in Figures 5B,C.
3. ln ε_max_: This corresponds to the largest value in the error growth curves, or the right most point in SDLE curves. Using an ensemble forecasting framework, we have proven that this quantify plays the role of the maximal amount of information (Gao et al., 2012c). The results are shown in Figure 5D.
4. SDLE_max_: the maximal SDLE value, shown in Figure 5E.
5. SDLE_ε^*^_: this is the SDLE at a fixed scale ε^*^. It plays a similar role as the commonly used approximate entropy and sample entropy (Gao et al., 2012c). The results are shown in Figure 5F.

In summary, for all the six metrics derived from SDLE analysis, there appears to be little difference between healthy control and CFS subjects. This appearance is easy to validate with simple statistical tests, as shown in Table 3 (*p*-values from a 2 tailed *t*-test).

**Table 3:** **Statistical tests on SDLE parameters during the TSST**.

	Δε_**max**_	γ_**1**_	γ_**2**_	lnε_**max**_	**SDLE_max_**	**SDLE**_ε^*^_
NF mean	−0.017	−0.34	−0.054	−2.74	0.26	0.23
NF std dev	0.026	0.069	0.051	0.44	0.045	0.098
CFS mean	−0.028	−0.34	−0.031	−2.70	0.26	0.26
CFS std dev	0.05	0.065	0.031	0.46	0.044	0.094
*p*-value	0.35	0.71	0.026	0.71	0.72	0.14

As can be seen in Table 3, none of the tests of the SDLE parameters to distinguish case from control groups are significant save one, and that would lose its significance after correction for multiple hypotheses using, e.g., a Bonferroni correction or a consideration of false discovery rate (FDR).

## 4. Discussion

CFS is a debilitating medical disorder withno known biomarker. Recent small-sample studies about possible cardiac irregularities (Naschitz et al., 2006; Boneva et al., 2007; Burton et al., 2009; Hurwitz et al., 2009; Hollingsworth et al., 2011; Miwa and Fujita, 2011; Newton et al., 2011; Rahman et al., 2011; Beaumont et al., 2012) have motivated us to carefully examine whether HRV in persons with CFS may be substantially different from that in healthy controls. Given that previous studies have already tried to quantify the difference between the HRV of healthy control and CFS subjects using standard HRV metrics that assume stationarity, we have chosen two completely different multiscale methods, AFA and SDLE, that do not assume stationarity to analyze HRV, and used them on data from healthy control and CFS subjects in a highly non-stationary environment. These two methods are fundamentally different, because AFA belongs to random fractal theory, while SDLE has its origin in deterministic chaos theory, although SDLE has been proven to also be able to characterize various types of random processes. Using AFA, the data suggests potential differences between the HRV of healthy control and CFS subjects prior to social stress tests, but the differences seem to be diminished during the test itself. The latter observation is further supported by SDLE analysis. Both observations may require further statistical validation and potentially larger sample sizes.

The differences observed in resting HRV between CFS and healthy controls (before the stress tests) is consonant with the observation that the complexity of HRV for CFS patients is reduced during sleep (Boneva et al., 2007). Furthermore, the differences in power spectral density of HRV seen by Boneva et al. (a greater loss of low frequency power in CFS vs. high frequency power) are consistent with a shifting of the Hurst parameter in CFS toward an anti-persistent regime seen in this study. While it might not be appropriate to conclude that there is no difference between the HRV of healthy control and CFS subjects during the TSST, the trend is clear—the difference between HRV of control and CFS subjects appears to be reduced during stress. This trend has also been suggested recently by other studies (Beaumont et al., 2012).

As we pointed out in the beginning, the purpose of our paper is twofold—to analyze HRV as a window into the potential ANS anomalies and pathophysiology of CFS, and to demonstrate the general use of AFA and SDLE for analyzing non-stationary HRV. The character of the stress test employed during this study naturally makes our HRV data more non-stationary than those measured in resting states. The results presented here suggest that AFA and SDLE could be very useful for the analysis of HRV, measured in both stationary and non-stationary environments.

### Conflict of interest statement

The authors declare that the research was conducted in the absence of any commercial or financial relationships that could be construed as a potential conflict of interest.
